# The “virgin birth”, polyploidy, and the origin of cancer

**DOI:** 10.18632/oncoscience.108

**Published:** 2014-12-17

**Authors:** Jekaterina Erenpreisa, Kristine Salmina, Anda Huna, Thomas R. Jackson, Alejandro Vazquez-Martin, Mark S. Cragg

**Affiliations:** ^1^ Latvian Biomedical Research & Study Centre, Riga; ^2^ Faculty Institute for Cancer Sciences, University of Manchester, Manchester Academic Health Science Centre, UK; ^3^ Southampton University School of Medicine, Southampton, UK

**Keywords:** cancer, embryonality, polyploidy, accelerated senescence, parthenogenesis

## Abstract

Recently, it has become clear that the complexity of cancer biology cannot fully be explained by somatic mutation and clonal selection. Meanwhile, data have accumulated on how cancer stem cells or stemloids bestow immortality on tumour cells and how reversible polyploidy is involved. Most recently, single polyploid tumour cells were shown capable of forming spheroids, releasing EMT-like descendents and inducing tumours *in vivo*. These data refocus attention on the centuries-old embryological theory of cancer. This review attempts to reconcile seemingly conflicting data by viewing cancer as a pre-programmed phylogenetic life-cycle-like process. This cycle is apparently initiated by a meiosis-like process and driven as an alternative to accelerated senescence at the DNA damage checkpoint, followed by an asexual syngamy event and endopolyploid-type embryonal cleavage to provide germ-cell-like (EMT) cells. This cycle is augmented by genotoxic treatments, explaining why chemotherapy is rarely curative and drives resistance. The logical outcome of this viewpoint is that alternative treatments may be more efficacious - either those that suppress the endopolyploidy-associated ‘life cycle’ or, those that cause reversion of embryonal malignant cells into benign counterparts. Targets for these opposing strategies are components of the same molecular pathways and interact with regulators of accelerated senescence.

“The aim of science is to seek the simplest explanations of complex facts. We are apt to fall into the error of thinking that the facts are simple because simplicity is the goal of our quest. The guiding motto in the life of every natural philosopher should be, ‘Seek simplicity and distrust it”

(Alfred North Whitehead, The Concept of Nature, 1929)

After more than 40 years of the “war on cancer” progress in achieving long-lasting cures and treating advanced, late stage, disease is still unsatisfactory. This failure likely stems from our limited understanding of the true complexity of the disease [1]. Attempts to define the basis behind cancer are many and varied, dating back centuries. One particular concept, the embryological theory of cancer, has existed for more than 150 years and was developed during the 19^th^ century by prominent scientists of that time [2, 3]. Amongst these, David von Hansemann, wrote in 1890 that normal somatic cells can undergo de-differentiation and transform into cancer cells, which acquire “egg-like” features [4].

The biological equivalency between embryos and tumours was experimentally established in 1964 by Leroy Stevens who showed that normal pluripotent embryonic stem cells from murine blastocysts, could develop into teratomas/teratocarcinomas if they were injected into an adult testis or into an embryo if injected back into a uterus [5]. The same year, Barry Pierce and colleagues demonstrated the ability of a single malignant teratocarcinoma cell to form a primitive embryoid body with the capacity to give rise to the three major germ-cell layers [6, 7], subsequently showing these embryonal properties for other carcinomas [8]. It was also shown that teratocarcinoma-embryo chimeras can be produced if the malignant cells are placed into the environment of a normal blastocyst [9, 10]. Although these experiments were forgotten for many years, in modern times induced pluripotent stem cells (iPSC) have been tested for their ability to cause teratomas and teratocarcinomas. Given all of these observations, and the current frustrations in our ability to understand the complexity of cancer and establish effective cures, even with our sophisticated ability to unpick their underlying somatic mutations and clonal architecture, it may be time to revisit the half-forgotten embryological theory of cancer.

The currently popular cancer stem cell (CSC) theory of tumourogenesis assigns the property of immortality (self-renewal) to adult stem cells that due to genetic mutations, or epigenetic changes, de-differentiate to a state similar to very early embryonal (ESC-like) cells [11, 12]. However, although this concept explains how proliferation capacity is extended, it does not explain why immortality is supported in tumours again and again. The only known natural process capable of supporting immortality indefinitely is the life cycle, which transfers the germ-line through one generation to the next. Recognition of this basic biological law formulated by August Weismann more than a century ago lies at the core of the embryological theory of cancer and its many variants [13].

Through more than a decade of research in our and other labs, it has been seen that meiotic genes are activated in TP53 mutant tumours, enhanced by genotoxic treatments or spindle inactivation and associated with reversible polyploidy capable of recovering clonogenic diploid cells [14-16]. Earlier induction of c-Mos by paclitaxel in SKOV3 cells was shown by Ling et al., [17], while Gorgoulis et al.,[18] found it in primary small cell lung cancer. Similarly, data on the presence of the so-called cancer testes-associated antigens (CTA) in tumours, among them meiotic, embryonal and placental gene products, revealed a link between gametogenesis and cancer [19, 20]. These data tempted Lloyd Old to provocatively entitle one of his commentaries “Cancer is a somatic cell pregnancy” [21]. He wrote: “Because many of the cardinal features of cancer are also characteristic of gametogenesis/placentation, e.g. migration, invasion, immune subversion, apoptosis resistance, induction of angiogenesis, etc., it takes little imagination to think that cancer-testes gene products controlling these processes during gametogenesis confer these same capacities on the cancer cell”. This statement contains a frank recognition of the embryological theory of cancer.

The last decade has added yet more complexity. In addition to activation of the main meiotic kinase Mos and genes of meiotic prophase, certain division features characteristic for meiosis (cohesion of sisters and omission of one S-phase) were observed in genotoxically-treated cancer cells with the involvement of the meiotic cohesin REC8 and recombinase DMC1[22]. Mos activation was also found during endomitosis [23] or multi-polar mitosis of TP53-mutant tumours[16, 24]. Our initial speculations on these data were that cancer is associated with a programmatic recapitulation of the ancient ploidy-cycles and asexual life-cycles of early protists [23, 25, 26]. Some researchers described somewhat similar changes, but did not associate them with meiosis. Formation of endo-tetraploid cells with diplochromosomes through cohesion of sisters as a consequence of DNA damage was substantiated by Davoli and Lange [27]. Walen described (in normal senescing or glutamine-deprived cells) a-spindled co-segregation in endotetraploid cells of entire (haploid) genome complements (2x2C1n) and suggested the significance of this process for carcinogenesis [28, 29]. However, further studies revealed that not only meiotic genes but also the key genes of early embryogenesis were induced by genotoxic treatments in various tumour cell lines, (both male and female), and associated with reversible polyploidy [30-32]. ESC-like gene signatures were also revealed in aggressive primary tumours [33-36] and it was shown that neoplastic non-stem cells could spontaneously convert into CSCs through epigenetic regulation [37-40].

Two important new aspects were subsequently added to our understanding: (1) as predicted by Blagosklonny (2007) differentiated tumour cells were shown to have the capacity to de-differentiate and become CSC (or “stemloids”) [41]; (2) induction of stemness by DNA or spindle damage was shown to be associated with the activation of meiotic genes coupled to reversible endopolyploidy (in TP53 function deficient cells). So, along with meiosis, life-cycle features were coupled to reversible polyploidy as evidenced by the ectopic ESC gene expression in somatic tumour cells. To unite these aspects, we proposed that cancer-related polyploidy appeared at the same point in macro-evolution at which multicellularity occurred (formed by reversible endopolyploidy) in unicellular organisms [42]. The rationale for this was because this evolutionary period coincides with the diversification of somatic and gametic lineages, simultaneous with the emergence of sexual reproduction and gastrulation [43].

While reversible polyploidisation of tumour cells through aborted mitoses (“mitotic slippage”) is now established [44, 45] currently, it is unclear how the giant polyploid tumour cells de-polyploidise. Various proposals have been made including; meiosis-like reduction divisions [15, 22]; reduction through diplochromosomes and haploidy [28, 29]; multi-polar mitoses [16, 46, 47]; and “a-mitotic budding” of descendent sub-cells [48-51]. Recently, we attempted to unite and order most of these various events as a necessary sequence of steps in a prolonged process of specific rearrangements [31, 52]. However, the mechanisms associated with survival of resistant tumour cells after the emergence of reversible polyploidy under genotoxic stresses remain hitherto ill-defined, at least in part because of the complexity, extension in time, and rarity of the process (as most cells die), and so the full picture remains obscure.

Furthermore, despite the substantial progress in understanding the crucial role of polyploidy in cancer, recently reviewed by Coward and Hardings [53], clearly, we are currently unable to answer many questions. For example: Is the meiosis-like response of TP53-mutant tumours to genotoxic treatments followed by any syngamy (fertilization-like) event(s)? If so, how and when do they take place?; Why is stemness induced in somatic tumour cells by DNA damage, and why is it associated with transient (reversible) polyploidy?;Why do the sub-nuclei of polyploid giants cells behave autonomously and undergo asymmetric divisions [31, 54, 55];How and why do multinucleated tumour cells sequestrate cytoplasm to their individual sub-nuclear descendants [31]?; How do they identify and sort the sub-nuclei containing viable or non-viable genetic material [31, 42, 55]?; Why does the extent of reversible polyploidisation have an apparent limit of ~32C which coincides with the cell number in the morula (blastulation) stage of embryogenesis [42] and are these somehow related?; Why is accelerated senescence coupled to induced stemness in these DNA damaged cells [52, 56-58]?; What explains the kinetics of MOS and REC8 activation during the polyploidisation and de-polyploidisation processes [14, 15]?

Most of these questions can potentially be addressed in terms of the parthenogenetic theory of cancer, which stemmed from the embryonal theory. We have occasionally seen visual evidence that could be interpreted in this way in our own studies; some examples of which are presented in Fig. 1.

**Fig.1:**
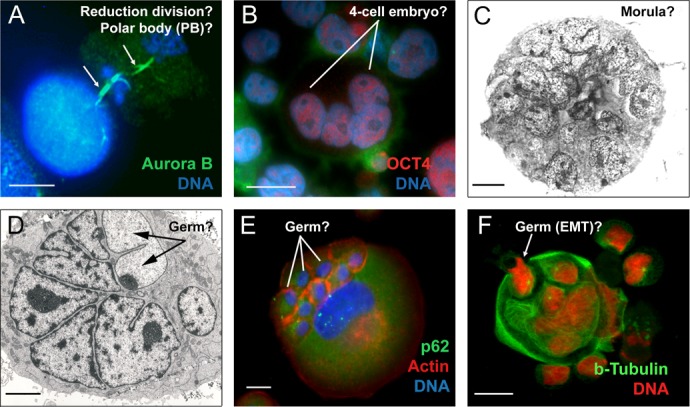
Embryonal features of endopolyploid tumour cells induced by DNA damage A) Namalwa cells (44, X, -Y) (post 10 Gy irradiation). Two asymmetric reduction divisions are observed which resemble the formation and subsequent division of the first polar body (PB) in the maturing oocyte (2 hour treatment with lactocystin before fixation allowed the preservation of both mid-bodies, arrowed), republished from [54]; B) WI-L2-NS (47, XY) cells (post 10 Gy irradiation). The induced giant polyploid cell resembles a 4-cell embryo; C) HeLa S3 (68, XXX) cell line (post 10 Gy irradiation). A tumour-spheroid that resembles a morula is observed; (republished from [15] ; D) Namalwa cells 14 days post 10 Gy irradiation. Transmission EM of a giant polyploid tumour cell following aborted radial division. Two types of subnuclei are observed; one with a conventional structure and another with a juvenile-like structure (not present in non-treated controls, arrowed); E) A431MetforR (metformin-resistant) cells were selected as indicated [59], (72, XX). A giant cell showing mild autophagic activity and actin-enriched individual cytoplasmic regions around small sub-nuclei; F) Namalwa polyploid giant cell budding a cellularised descendant that has been sequestered from the parent's cytoplasm 13 days post 10 Gy irradiation, republished from [26]. Bars =10 μm.

Parthenogenesis *(“virgin birth”* in Greek) is a special reproductive strategy widely used in the plant and animal kingdom, in which an unfertilized egg is reunited with a polar body, and undergoes embryogenesis to develop adult offspring. The parthenogenetic theory of cancer was first suggested by Beutner [60] (cited from Erenpreiss [2]) and updated more recently by Vladimir Vinnitsky [3, 61]. This link is also made more apparent with a series of recent studies reporting the spherogenicity and malignancy of endopolyploid tumour cells (ETC). In these experiments, polyploid giant cancer cells were sorted either manually [62], or chemically – using the hypoxiamimic CoCl_2_ [50, 63] or by serial selections in etoposide [51]. These ETC displayed increased resistance to chemo-radiotherapy, expressed key ESC and germline factors (Oct4/Nanog, Sox2, SCF, c-kit), and surface markers (CD44, CD133) as well as an ESC-like microRNA profiles. These single ETC were shown capable of forming tumour spheroids which could undergo differentiation into the three germ layers and critically to form tumours in immunodeficient mice with high efficiency [50, 62]. In other words, the revelations of Barry Pierce and colleagues detailed earlier for single carcinoma cells have now been shown to be attributable to single ETC. These experiments were performed on tumour cell lines representing almost all cancer types (breast, ovarian, bladder, colon, glioblastoma, fibrosarcoma, osteosarcoma, retinoblastoma, lymphoma). Moreover, it was shown that these giant polyploid tumour cells possessing large subnuclei ultimately bud smaller cells [51] of fibroblastic shape and with markers of epithelial-mesenchymal transition (EMT) [50]. The occurrence of asymmetric mitotic divisions in the late ETC which precede cellularisation and the release of rejuvenated sub-cells was also suggested by us previously [31]. Thus, through the generation and reversal of polyploidy coupled to this embryonal-type stemness induction, these tumour cells potentially elicit an “invasion” phenotype in their descendants. Theoretically and based on our cancer cell “life cycle” hypothesis wherein reversible polyploidy releases the germline [24-26] it means that the cells undergoing EMT with ‘embryo-like” features are the biological equivalent of a germ cell, as also concluded by Zhang and colleagues [50].

These observations and conclusions largely fit the embryonal theory of cancer. Its oncogerminative variant is proposed by Vladimir Vinnitsky [3, 61] and illustrated in Fig.2. Within the scheme, three main tenets are outlined: reproduction of the oncogerminative cell by an embryonal cleavage-like process (with the parthenogenetic origin of the tumour initiating CSC); the equivalence between the tumour spheroid and the a-vascular blastocyst-stage of embryogenesis; and the invading potential of the germline (EMT) mimicking the biological properties of primordial germ cells (PGC) in normal embryogenesis. The similarity between PGC and migrating tumour cells was previously supposed by John Beard in 1902 [64], cited from Beckett [65] highlighting the embryological theory as a gateway to the cancer stem cell theory. Notably, cycles of MET-EMT epigenetic transitions interspersed by this embryonal life-cycle are proposed by Vinnitsky as the mechanism behind the observed ongoing cancer relapses.

**Fig.2:**
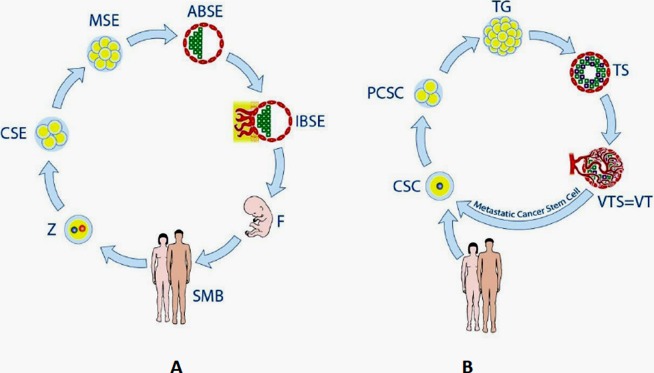
The figure and legend are reproduced from [3], with consent of Vladimir Vinnitsky Stages of the life cycles of germline cells (A) and oncogerminative cells (B). (A) Z, zygote; CSE, cleavage stage embryo; MSE, morula stage embryo; ABSE, avascular blastocyst-stage embryo; IBSE, implanted blastocyst-stage embryo; F, fetus. SMB, sexually mature body. (B) CSC, cancer stem cell (i.e., oncogerminative cell); PCSC, parthenogenetic cancer stem cell (a pseudo-cleavage-stage embryo); TG, tumor germ (a morula-stage embryo-like structure); TS, tumor spheroid (an imitation avascular blastocyst-stage embryo); VTS/VT, vascularized tumor spheroid and/or vascularized tumor (an implanted blastocyst-stage embryo-like entity).

Although Vinnitsky did not consider the polyploidisation of tumour cells as a participant in this embryological process, the very idea of parthenogenesis provides a place for the observed activation of meiotic genes and meiotic-like divisions in the DNA damaged tumour cells because parthenogenesis needs first formation and maturation of an oocyte. Moreover, the main driver of oogenesis, Mos-kinase, was shown to be induced by genotoxic treatments in tumour cells of various origins, as described above. Therefore, a somatic meiosis-like process* seems to be the first step in the DNA damage response. Mos can also arrest cells in a ‘mitotic checkpoint” protecting them from apoptosis as an alternative to mitotic catastrophe [24]. Given the evidence outlined above relating to ETC, the polyploid giant cells appear to represent pathological analogs of the early embryo. Subsequently, a syngamic event is required to occur between “meiosis” and “embryo cleavage”. A hypothetical scheme of how these key events may occur in sequence is presented in Fig.3.

**Fig.3:**
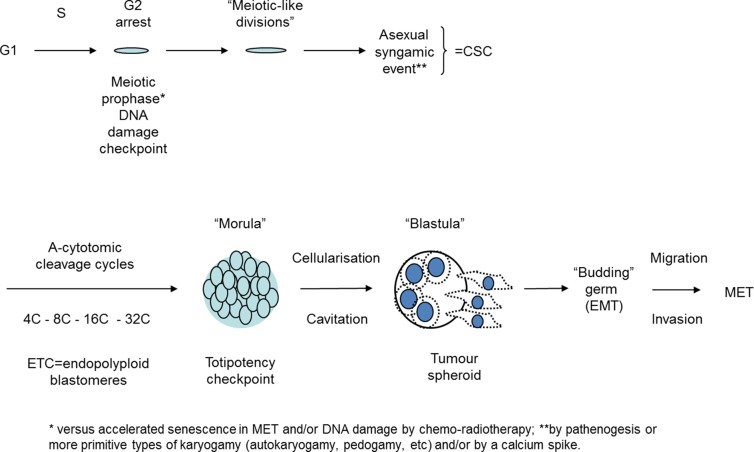
Hypothetical scheme of cancer cell ‘life cycle’ based on embryological concept and experimental observations of TP53 dysfunctional tumour cell lines after genotoxic treatments

* somatic meiosis does not necessarily need recombination between homologs, which may be substituted by recombination between sister chromatids [66].

A polyploid giant cell leading to the formation of a tumour spheroid, when put into the context of the embryological process (coined a pseudo-morula and pseudo-blastocyst by Vinnitsky 2014), coincides with our previous notion that the development of a giant tumour cell (through endopolyploidisation) is equivalent to the embryonal cleavage (endo-)cycles that reach “a developmental checkpoint of totipotency” at 32C. This represents ~4 abortive mitoses, which occur by day 5, the point from which the reverse of polyploidy, ultimately ending in de-polyploidisation and release of mitotic descendants, is initiated [31, 55, 67]. This period of five days may represent a checkpoint of tumour cell endopolyploidisation of embryonal origin and is seen in several other models [32, 68]. Here, we should explain why tumour ‘pseudo-blastomeres’ are polyploid. During cleavage of the tumour pseudo-embryo the pseudo-blastomeres do not undergo full cytotomy until the blastula-equivalent stage, after which the giant cell sub-nuclei undergo cellularisation (sequestration of individual cytoplasm territories) preceding the ‘budding’ of daughter cells (described in [26, 31, 48, 50, 51]. This type of cleavage where a syncytial blastoderm is formed and shortly afterwards a cluster of germ cells is separated along with cellularisation is well-characterised in Drosophila, perhaps the best understood of all developmental systems [69]. Endopolyploidy, multi-nucleation with multiplication of pronuclei and/or polar bodies are seen in pathological human eggs (numerous examples are seen in the pictures from the Advanced Fertility Center of Chicago http://www.advancedfertility.com/abnormal-ivf-egg-pictures.htm). These pictures resemble those of multi-nucleated (“pregnant”) giant tumour cells presented by Diaz-Carballo, et al. [51] and Zhang, et al. [50]. Interesting changes are observed in giant tumour cells after reaching “the totipotency checkpoint”. Radial divisions (incomplete cytotomy) described previously by us in late ETC occur which precede casting off the external layer of cytoplasm along with cellularisation and release of small descendants by ‘budding’ [31, 44, 67]. In some way, this process is associated with diversification (by asymmetric divisions) of the giant cell sub-nuclei into two types, larger (not individualizing cytoplasm) destined to degenerate, and smaller (proliferative, endowed by stemness factors and decompacted chromatin, acquiring their own cytoplasm and actin ring) for shedding with the aid autophagy (Fig. 1D-F). Radial cleavage furrows is a well-known feature of the animal cleavage pattern which situates the blastomeres in a doughnut pattern where they remain totipotent and bridged up to the 32 cell stage as reported in Volvox [70]. Curiously, fossils of early animals dated ca. 570-620 Ma were found to show 32-cell blastula [71]. The genealogy of cancer may therefore be truly ancient!

However, there are two key points in the scheme of Vinnitsky that require further clarification. 1) How do settled MET cells return to the parthenogenetic life-cycle again, and what drives their entry/exit; 2) what is the role of the reversible accelerated senescence reported by multiple observers [58, 68, 72-75] which accompanies reversible polyploidy in this process and paradoxically can favour survival? The latest data demonstrate that tumour cells can shuttle between low and high malignant states according to their MET/EMT status through epigenetic changes of bi-stable chromatin [40]. This addresses the biochemical aspects of the phenomenon. Here, we would like to suggest that biologically the MET cells, previously produced from EMT, may undergo accelerated cellular senescence due to accumulation of ROS and DNA strand breaks. The accumulation of DNA damage is a hallmark of accelerated senescence [76]. However, DNA damage is also considered as a major cause for the evolution of DNA repair by recombination, which gave rise to meiosis through ploidy cycles [23, 66, 77]. Interestingly, a simple doubling in the amount of ROS is sufficient to induce the genes which initiate the facultative sexual reproduction cycle in Volvox *carteri* [78], indicating the ancestral role of sexual reproduction as an adaptive response to stress and ROS-induced DNA damage [77].

Markers of accelerated senescence and DNA damage were unexpectedly found to be linked to markers of stemness in senescing IMR-90 fibroblasts [56] and etoposide-or irradiation treated tumour cells arrested in G2-phase [57, 58]. The p21-dependent senescence related to stemness pathways (TGF-β and PI3K) was revealed in normal embryogenesis [79]. Moreover, senescence markers are predictors of poor prognosis in lung cancer patients after neo-adjuvant therapy [74]. All of this highlights the close link between accelerated senescence and stemness and their relationship to carcinogenesis [52].

The origin of sexual reproduction is a complex and much debated issue. DNA recombination by meiosis coupled with sex (i.e. the fusion of paternal gametes), although costly in energetic terms, apparently provides the optimal balance between DNA repair and genetic variation [80]. Polyploidy as such provides the advantage of masking deleterious mutations [66], resistance to toxicity and energy depletion [81] and therein better survival of polyploid tumour cells in unfavourable conditions [53]. Reversible polyploidy coupled with the generation of immortal germ cells therefore captures the advantages of both. We propose that mammalian cancer cells use exactly this phylogenetic program ensuring immortality (by a life-cycle) and genome plasticity and heterogeneity (by stemness) through epigenetic and genetic variation (and subsequent clonal selection) as a basis for developing resistance to treatments.

It can be suggested that accelerated senescence of genotoxically damaged tumour cells serves as a bridge and stochastic option for the initiation of the next (cancer) life-cycle, which is also accelerated (starting from meiotic- and syngamic-like events and ending with the blastula/spheroid through formation of the germ-like EMT tumour cells in a process akin to early embryogenesis carried out in approximately one-two weeks). Interestingly, our recent research on etoposide-treated embryonal carcinoma cells showed that a potential regulator and trigger for this switch is OCT4 (POU5F1), a carrier of life-cycle totipotency [82]; and induced in a TP53-dependent manner alongside p21CIP1. The choice between the two opposite cell fates (reinitiate cell divisions or undergo terminal senescence) in transiently bi-potential cells is undertaken in G2 arrest [57] and this barrier can become adapted to start polyploidy [56]. When the tetraploidy barrier is overcome, the TP53 tumour-suppressing function becomes surpassed [83], likely by methylation of its promoter [84], while mTOR linking to p21-mediated senescence becomes suppressed, thus allowing the reversal of senescence [58]. We suggest that the cancer cell ‘life cycle’ is therefore initiated by meiosis and locked by accelerated senescence with the opposing outcomes diverging from the same DNA damage checkpoint [52] supporting the earlier supposition that “Carcinogenesis always is started with immortalisation. That is a possibility to overcome senescence” [2, 85]. We therefore arrive at the same conclusion suggested by Rajaraman [49] that immortality of cancer cells is not perpetual but becomes cyclically renewed.

Intriguingly, an ~5 day period of ‘stochastic’ choice between senescence/MET and self-renewal/EMT, separated by a longer rate-limiting period of further determination of self-renewal has also been reported during the induction of pluripotent stem cells (iPSC) [86], with the whole process taking ~30 days.

One further consideration of the parthenogenetic origin of cancer is that parthenogenesis is typically viewed as a female privilege. Although commonly believed, this is not quite true. Mammalian primordial germ cells (PGC) of either XX or XY karyotype are sexually dimorphic and have the potential to enter either spermatogenesis or oogenesis. In a female genital ridge, or in a non-gonadal environment *in vivo*, as well as autonomously in tissue culture both 46XX and 46XY PGCs develop as meiotic oocytes [87] and can therefore initiate parthenogenesis. Only male gonadal somatic cells, which differentiate, through SRY (sex determining gene on Y chromosome) instruction to SOX9, into Leydig and Sertoli cells producing and stabilizing testosterone inhibiting PGCs from entering oogenesis, are directed to a spermatogenic fate [88]. Therefore, it should be no surprise that male ECS cells can undergo development until the blastula stage as described by Hubner and Scholer[89] and that male lymphoblastoma cells, like WI-L2-NS, induce key drivers of oogenesis such as Mos[14], and OCT4 [30] and can develop after genotoxic damage into early embryo-like OCT4-positive giant cells (Fig.1B). Interestingly, there are reports of the frequent loss of Y chromosomes in male cancers, for example at initial diagnosis of myeloid malignancies, with restoration of the normal karyotype (46XY) at remission [90].

An additional facet to be considered relates to aneuploidy. Aneuploidy is a typical hallmark of cancer, accompanying polyploidy and is usually explained by the instability of the cancer genome losing or gaining chromosomes during proliferation [91, 92]. However, this approach does not explain the “aneuploidy paradox”: given its inherent anti-proliferative potential, why is aneuploidy obstinately retained in proliferating tumours [93, 94]?. Why do many cancers keep to para-triploidy, which in childhood neuroblastoma is associated with less chromosomal aberration and more favourable outcome [95], while other tumours are para-haploid [28, 96]? This paradox may be also resolved within the parthenogenetic theory of cancer.

Animals use multiple mechanisms of parthenogenesis, most of which depend on polar bodies that do not degenerate. So, besides several variants of parthenogenetic fusions leading to restoration of 2n chromosome number, there are also other variants allowing triploid cells to form [97]. Notably, ESCs resulting from haploid mouse parthenogenesis can also acquire germline potency after diploidisation [98], which can thus lead to loss of heterozygosity and loss of tumour suppression. The essence of meiosis is genetic recombination, so that each meiotic division produces a distinct sibling. Embryos that retain their polar bodies during development thus have two or more different genomes enabling genetic variation a-sexually. If tumour cells are involved in a macroevolutionary predetermined life-cycle with parthenogenetic components, then segregation of haploid paternal genomes and their variable combinations during the parthenogenetic fusions may be nearly an inevitable component of this process recovering the immortal germ-line. In turn, losses and gains of separate chromosomes are only secondary acquisitions of tumour microevolution, driven by instability and stochastic events, which may be counterbalanced by the recombinative process of meiosis [24]. Interestingly, the “triploid bridge” has been established in plants as facilitating a shift from polyploid to diploid generations [99].

## CONCLUSION

Accumulating data favours a change to the view of the phylo-ontogenetic origin of cancer as a pre- programmed life-cycle process. This view provides a conceptual framework within which to explain the origin of cancer cells, their immortality and resistance to genotoxic treatments, and allows resolution of much of the complexity behind cancer phenomenology. It furthermore allows us to place the current knowledge on CSC, the reversible polyploidy of giant polyploid tumour cells, and the ectopic induction of meiosis-like processes into a non-contradictory hypothesis.

### Perspectives

Accepting this concept of cancer cell embryonality and its life-cycle-like process of immortality provides us with new ways of understanding and treating cancer. On one hand, the embryonal-type plasticity and heterogeneity allows tumour cells to bypass many targeted therapies by substituting them with alternatives and thereby allowing time to undergo genetic drift and clonal selection [100, 101]. Moreover, genotoxic treatments actually favour the embryonalisation of tumour cells, thus promoting resistance, relapse and metastases [102-105] causing them to become entrapped in the embryonal “cancer attractor’ [106-108]. Therefore, truly targeted therapy should be designed to hit the ontogenetic root of the cancer life-cycle and maybe even its phylogenetic origin in early multicellularians [26, 42].

The view that the genes of cellular cooperation that evolved with multicellularity about a billion years ago are the same genes that malfunction to cause cancer is positioned as the atavistic theory of cancer, which is substantiated from both paleontology and genetics [109]. It is supposed that this program became suppressed in advanced Metazoans by newer genes, which are undoubtedly tumour suppressors. Multiple atavistic theories did not take into account the polyploidy component, however, we suggested previously and substantiated here that reversible polyploidy is an essential component of this evolutionary-originated cancer cell “life-cycle” program. This consideration brings us to the c-myc protooncogene, whose overexpression uncouples DNA replication from mitosis, thus leading to endopolyploidy [110]. C-myc is one of the most ancient genes of early Metazoans [111], linked during evolution to the Warburg effect [112]. It is also the main oncogene imposing immortality to cancer cells and a master regulator of stemness [52]. Importantly, c-myc is a gene, whose suppression in *in vivo* models eliminates “oncogene addiction” and cures experimental cancer [113, 114]. Therefore, the targets for interrupting the cancer cell ‘life-cycle’ at its evolutionary root should likely focus around c-myc.

On the other hand, if a tumour cell can undergo an epigenetic embryonalisation, its epigenetic reverse to a differentiated cell should be also possible. This strategy - not to fight but to tame tumour cells-seems logical. Such studies have been undertaken since the beginning of the 20^th^ century; (for rev. see [8, 115-117]. Recently, a very interesting experiment was performed showing that paclitaxel could induce both EMT and formation of benign fibroblasts in an ovarian cancer model [118]. The best known and widely applied example of this treatment strategy is the differentiation inducer all-trans-retinoic acid (RA). Intriguing this is an old Chinese medicine against cancer and capable of curing acute myeloid leukemia [119].

Tumours can be “normalized” by an embryonic morphogenetic field [120] or by putting them within a normal 3D stroma [121, 122]. The influence of a regenerative environment was seen by the insertion of sarcoma cells into a fractured rat tibia; the cartilage calus formation enslaved them and interrupted their invasive growth [123].

The most important thing is that while genotoxic treatments convert malignant tumour cells into even more malignant variants [105], the opposite strategy may convert a malignant tumour into a benign one and prevent metastases. The epigenetic reprogramming of tumor cells by inducing differentiation (f.ex. by cytokines) show that epigenetics wins over genetics [124]. This facet in principal confirms the notion that embryonalisation is the only essential biological feature of tumour cells [2, 42, 107].

The experiments with nuclear cloning of embryonal carcinoma cells revealed that both malignant and embryological potentials can co-exist [125]. Therefore although it may be impossible to obtain the irreversible normalization of genotypically altered tumour cells by epigenetic means [126], it should be possible to stop tumour progression [117].

However, the most exciting thing is that the potential targets for these opposing strategies, as well as the pathways for genotoxically induced resistance and accelerated senescence, all converge at the same molecular pathways, around c-myc. Suppression of Wnt/β-catenin signalling (which up-regulates c-myc to promote cell proliferation), favours the RA-dependent differentiation of embryonal carcinoma [127]. Potential normalization targets in spontaneous TP53 mutant tumour revertants lead to, among others, presenilin1 activating Notch1 substrate γ-secretase, up-stream of c-myc stress signaling [117]. In turn, Notch1, which directly regulates c-myc is co-operating with Wnt in enhancing tumorigenesis [128] enriches mammospheres induced in breast cancer by irradiation [129]. In addition, it was also found that p21CIP1, involved in regulation of cellular senescence, functions as a negative transcriptional regulator of WNT4 downstream of Notch 1 [130] and that p21CIP1 potentially reorganizes the nucleus during tumour reversion [117]. So, at the molecular level, all roads meet. This provides the hope that a single key, unavoidable, pathway may be targeted to finally cure cancer.
